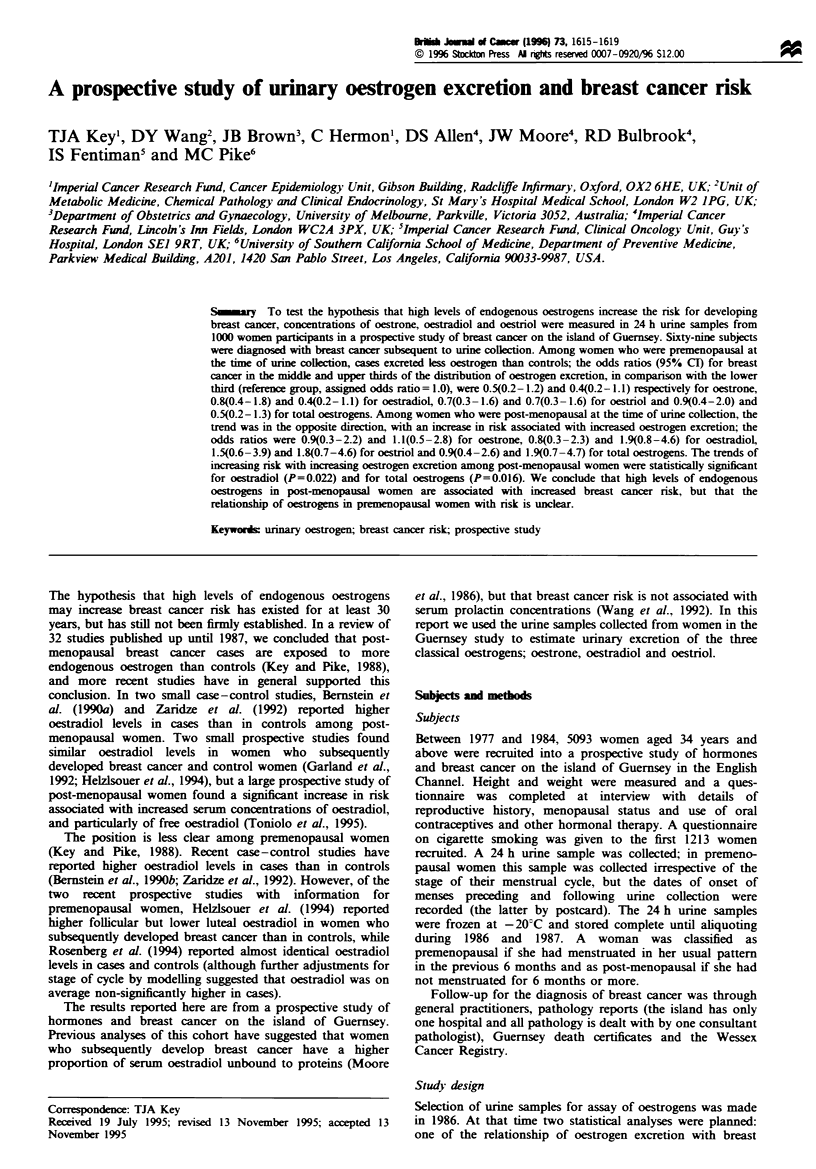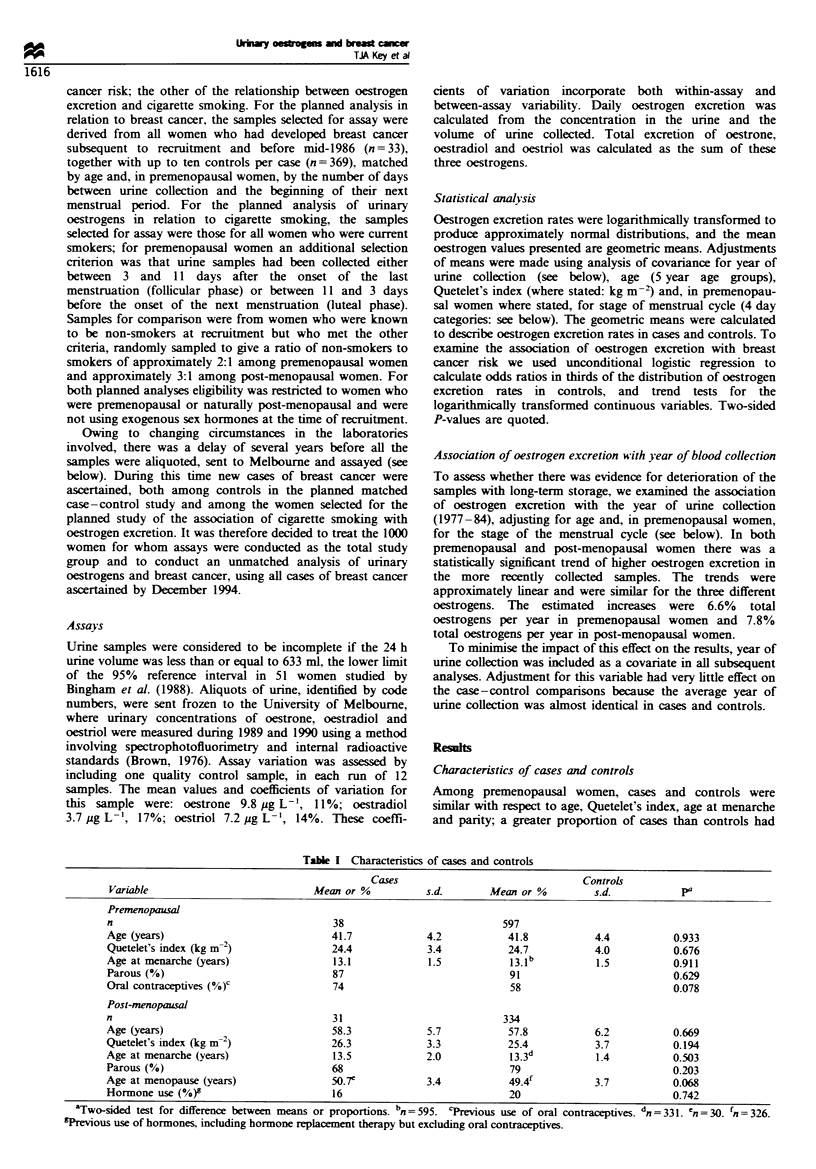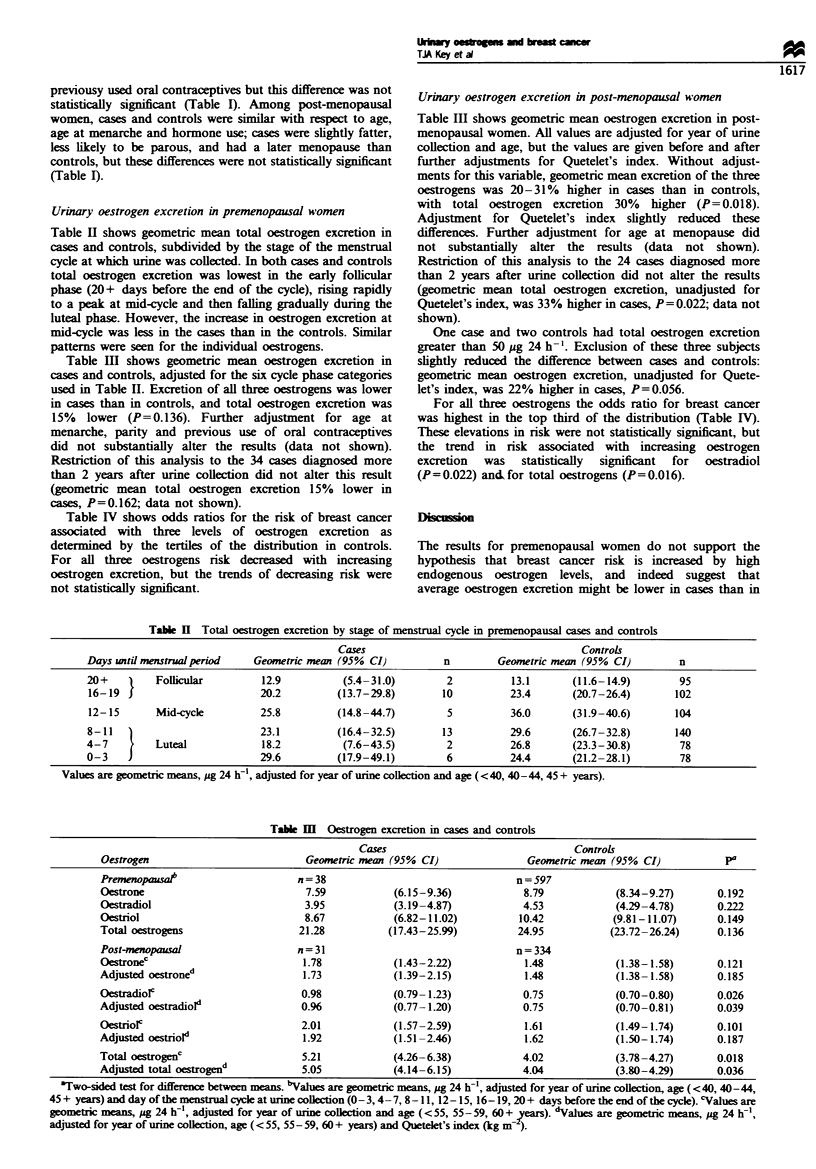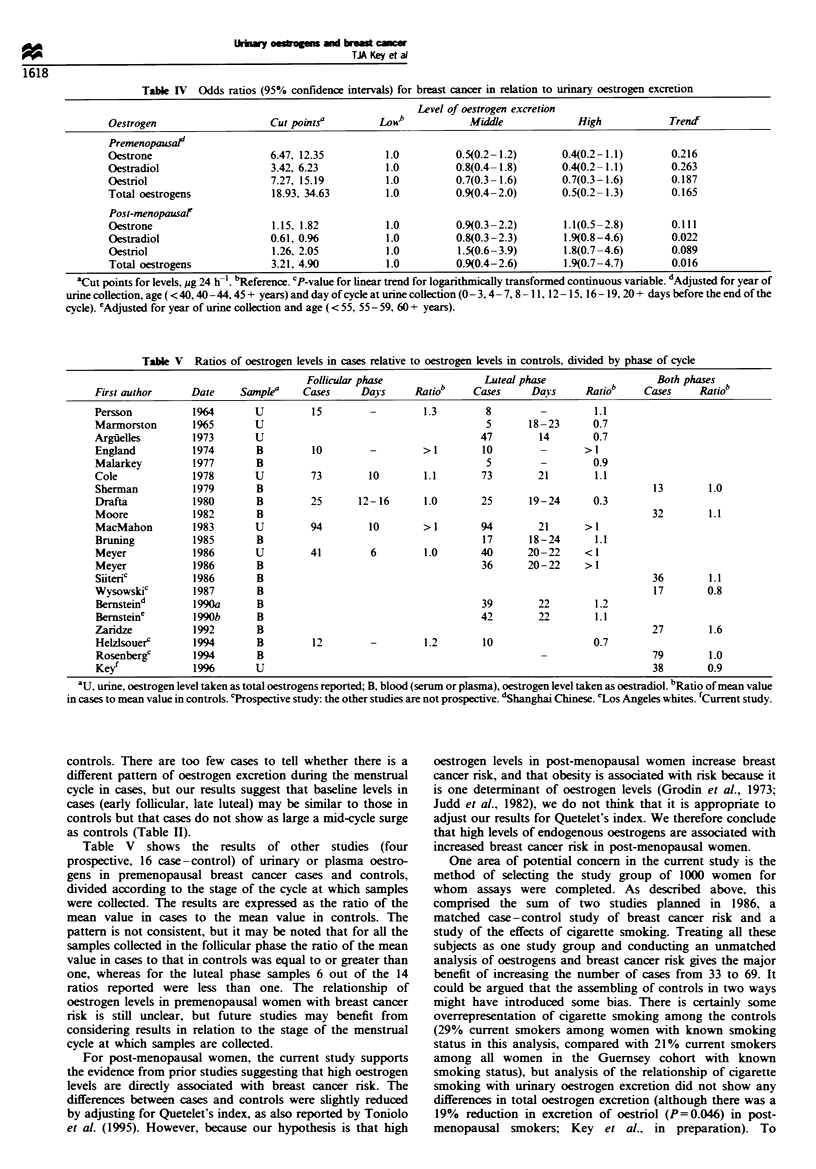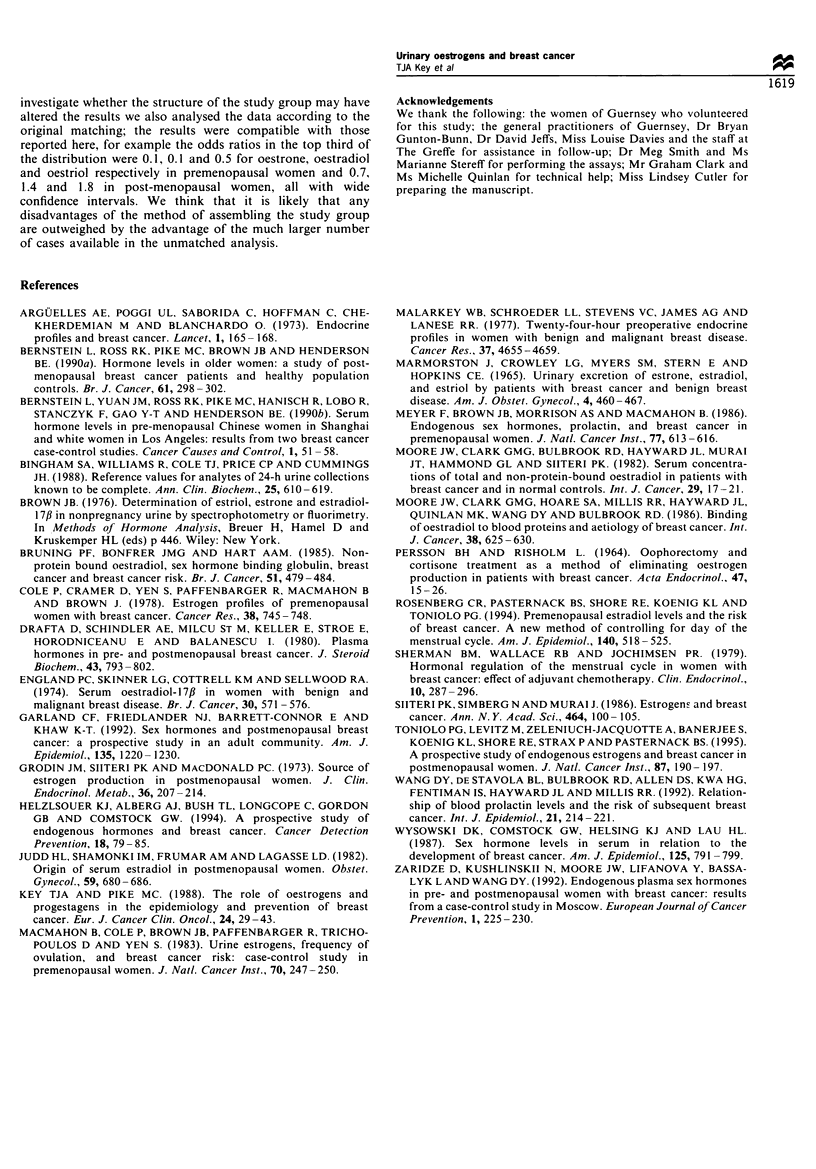# A prospective study of urinary oestrogen excretion and breast cancer risk.

**DOI:** 10.1038/bjc.1996.304

**Published:** 1996-06

**Authors:** T. J. Key, D. Y. Wang, J. B. Brown, C. Hermon, D. S. Allen, J. W. Moore, R. D. Bulbrook, I. S. Fentiman, M. C. Pike

**Affiliations:** Imperial Cancer Research Fund, Cancer Epidemiology Unit, Radcliffe Infirmary, Oxford, UK.

## Abstract

To test the hypothesis that high levels of endogenous oestrogens increase the risk for developing breast cancer, concentrations of oestrone, oestradiol and oestriol were measured in 24 h urine samples from 1000 women participants in a prospective study of breast cancer on the island of Guernsey. Sixty-nine subjects were diagnosed with breast cancer subsequent to urine collection. Among women who were premenopausal at the time of urine collection, cases excreted less oestrogen than controls; the odds ratios (95% CI) for breast cancer in the middle and upper thirds of the distribution of oestrogen excretion, in comparison with the lower third (reference group, assigned odds ratio = 1.0), were 0.5(0.2-1.2) and 0.4(0.2-1.1) respectively for oestrone, 0.8(0.4-1.8 and 0.4(0.2-1.1) for oestradiol, 0.7(0.3-1.6) and 0.7(0.3-1.6) for oestriol and 0.9(0.4-2.0) and 0.5(0.2-1.3) for total oestrogens. Among women who were post-menopausal at the time of urine collection, the trend was in the opposite direction, with an increase in risk associated with increased oestrogen excretion; the odds ratios were 0.9(0.3-2.2) and 1.1(0.5-2.8) for oestrone, 0.8(0.3-2.3) and 1.9(0.8-4.6) for oestradiol, 1.5(0.6-3.9) and 1.8(0.7-4.6) for oestriol and 0.9(0.4-2.6) and 1.9(0.7-4.7) for total oestrogens. The trends of increasing risk with increasing oestrogen excretion among post-menopausal women were statistically significant for oestradiol (P = 0.022) and for total oestrogens (P = 0.016). We conclude that high levels of endogenous oestrogens in post-menopausal women are associated with increased breast cancer risk, but that the relationship of oestrogens in premenopausal women with risk is unclear.


British Journal of Cancer (1996) 73, 1615–1619

© 1996 Stockton Press All rights reserved 0007-0920/96 $12.00

### A prospective study of urinary oestrogen excretion and breast cancer risk

TJA Key[1], DY Wang[2], JB Brown[3], C Hermon[1], DS Allen[4], JW Moore[4], RD Bulbrook[4], IS Fentiman[5] and MC Pike[6]

[1]*Imperial Cancer Research Fund, Cancer Epidemiology Unit, Gibson Building, Radcliffe Infirmary, Oxford, OX2 6HE, UK;* [2]*Unit of Metabolic Medicine, Chemical Pathology and Clinical Endocrinology, St Mary's Hospital Medical School, London W2 1PG, UK;* [3]*Department of Obstetrics and Gynaecology, University of Melbourne, Parkville, Victoria 3052, Australia;* [4]*Imperial Cancer Research Fund, Lincoln's Inn Fields, London WC2A 3PX, UK;* [5]*Imperial Cancer Research Fund, Clinical Oncology Unit, Guy's Hospital, London SE1 9RT, UK;* [6]*University of Southern California School of Medicine, Department of Preventive Medicine, Parkview Medical Building, A201, 1420 San Pablo Street, Los Angeles, California 90033-9987, USA.*

**Summary** To test the hypothesis that high levels of endogenous oestrogens increase the risk for developing breast cancer, concentrations of oestrone, oestradiol and oestriol were measured in 24 h urine samples from 1000 women participants in a prospective study of breast cancer on the island of Guernsey. Sixty-nine subjects were diagnosed with breast cancer subsequent to urine collection. Among women who were premenopausal at the time of urine collection, cases excreted less oestrogen than controls; the odds ratios (95% CI) for breast cancer in the middle and upper thirds of the distribution of oestrogen excretion, in comparison with the lower third (reference group, assigned odds ratio = 1.0), were 0.5(0.2–1.2) and 0.4(0.2–1.1) respectively for oestrone, 0.8(0.4–1.8) and 0.4(0.2–1.1) for oestradiol, 0.7(0.3–1.6) and 0.7(0.3–1.6) for oestriol and 0.9(0.4–2.0) and 0.5(0.2–1.3) for total oestrogens. Among women who were post-menopausal at the time of urine collection, the trend was in the opposite direction, with an increase in risk associated with increased oestrogen excretion; the odds ratios were 0.9(0.3–2.2) and 1.1(0.5–2.8) for oestrone, 0.8(0.3–2.3) and 1.9(0.8–4.6) for oestradiol, 1.5(0.6–3.9) and 1.8(0.7–4.6) for oestriol and 0.9(0.4–2.6) and 1.9(0.7–4.7) for total oestrogens. The trends of increasing risk with increasing oestrogen excretion among post-menopausal women were statistically significant for oestradiol ($P = 0.022$) and for total oestrogens ($P = 0.016$). We conclude that high levels of endogenous oestrogens in post-menopausal women are associated with increased breast cancer risk, but that the relationship of oestrogens in premenopausal women with risk is unclear.

**Keywords:** urinary oestrogen; breast cancer risk; prospective study

The hypothesis that high levels of endogenous oestrogens may increase breast cancer risk has existed for at least 30 years, but has still not been firmly established. In a review of 32 studies published up until 1987, we concluded that post-menopausal breast cancer cases are exposed to more endogenous oestrogen than controls (Key and Pike, 1988), and more recent studies have in general supported this conclusion. In two small case–control studies, Bernstein *et al.* (1990*a*) and Zaridze *et al.* (1992) reported higher oestradiol levels in cases than in controls among post-menopausal women. Two small prospective studies found similar oestradiol levels in women who subsequently developed breast cancer and control women (Garland *et al.*, 1992; Helzlsouer *et al.*, 1994), but a large prospective study of post-menopausal women found a significant increase in risk associated with increased serum concentrations of oestradiol, and particularly of free oestradiol (Toniolo *et al.*, 1995).

The position is less clear among premenopausal women (Key and Pike, 1988). Recent case–control studies have reported higher oestradiol levels in cases than in controls (Bernstein *et al.*, 1990*b*; Zaridze *et al.*, 1992). However, of the two recent prospective studies with information for premenopausal women, Helzlsouer *et al.* (1994) reported higher follicular but lower luteal oestradiol in women who subsequently developed breast cancer than in controls, while Rosenberg *et al.* (1994) reported almost identical oestradiol levels in cases and controls (although further adjustments for stage of cycle by modelling suggested that oestradiol was on average non-significantly higher in cases).

The results reported here are from a prospective study of hormones and breast cancer on the island of Guernsey. Previous analyses of this cohort have suggested that women who subsequently develop breast cancer have a higher proportion of serum oestradiol unbound to proteins (Moore *et al.*, 1986), but that breast cancer risk is not associated with serum prolactin concentrations (Wang *et al.*, 1992). In this report we used the urine samples collected from women in the Guernsey study to estimate urinary excretion of the three classical oestrogens; oestrone, oestradiol and oestriol.

Correspondence: TJA Key
Received 19 July 1995; revised 13 November 1995; accepted 13 November 1995

### Subjects and methods

### *Subjects*

Between 1977 and 1984, 5093 women aged 34 years and above were recruited into a prospective study of hormones and breast cancer on the island of Guernsey in the English Channel. Height and weight were measured and a questionnaire was completed at interview with details of reproductive history, menopausal status and use of oral contraceptives and other hormonal therapy. A questionnaire on cigarette smoking was given to the first 1213 women recruited. A 24 h urine sample was collected; in premenopausal women this sample was collected irrespective of the stage of their menstrual cycle, but the dates of onset of menses preceding and following urine collection were recorded (the latter by postcard). The 24 h urine samples were frozen at −20°C and stored complete until aliquoting during 1986 and 1987. A woman was classified as premenopausal if she had menstruated in her usual pattern in the previous 6 months and as post-menopausal if she had not menstruated for 6 months or more.

Follow-up for the diagnosis of breast cancer was through general practitioners, pathology reports (the island has only one hospital and all pathology is dealt with by one consultant pathologist), Guernsey death certificates and the Wessex Cancer Registry.

### *Study design*

Selection of urine samples for assay of oestrogens was made in 1986. At that time two statistical analyses were planned: one of the relationship of oestrogen excretion with breast cancer risk; the other of the relationship between oestrogen excretion and cigarette smoking. For the planned analysis in relation to breast cancer, the samples selected for assay were derived from all women who had developed breast cancer subsequent to recruitment and before mid-1986 ($n=33$), together with up to ten controls per case ($n=369$), matched by age and, in premenopausal women, by the number of days between urine collection and the beginning of their next menstrual period. For the planned analysis of urinary oestrogens in relation to cigarette smoking, the samples selected for assay were those for all women who were current smokers; for premenopausal women an additional selection criterion was that urine samples had been collected either between 3 and 11 days after the onset of the last menstruation (follicular phase) or between 11 and 3 days before the onset of the next menstruation (luteal phase). Samples for comparison were from women who were known to be non-smokers at recruitment but who met the other criteria, randomly sampled to give a ratio of non-smokers to smokers of approximately 2:1 among premenopausal women and approximately 3:1 among post-menopausal women. For both planned analyses eligibility was restricted to women who were premenopausal or naturally post-menopausal and were not using exogenous sex hormones at the time of recruitment.

Owing to changing circumstances in the laboratories involved, there was a delay of several years before all the samples were aliquoted, sent to Melbourne and assayed (see below). During this time new cases of breast cancer were ascertained, both among controls in the planned matched case–control study and among the women selected for the planned study of the association of cigarette smoking with oestrogen excretion. It was therefore decided to treat the 1000 women for whom assays were conducted as the total study group and to conduct an unmatched analysis of urinary oestrogens and breast cancer, using all cases of breast cancer ascertained by December 1994.

### *Assays*

Urine samples were considered to be incomplete if the 24 h urine volume was less than or equal to 633 ml, the lower limit of the 95% reference interval in 51 women studied by Bingham *et al.* (1988). Aliquots of urine, identified by code numbers, were sent frozen to the University of Melbourne, where urinary concentrations of oestrone, oestradiol and oestriol were measured during 1989 and 1990 using a method involving spectrophotofluorimetry and internal radioactive standards (Brown, 1976). Assay variation was assessed by including one quality control sample, in each run of 12 samples. The mean values and coefficients of variation for this sample were: oestrone 9.8 $\mu g\ L^{-1}$, 11%; oestradiol 3.7 $\mu g\ L^{-1}$, 17%; oestriol 7.2 $\mu g\ L^{-1}$, 14%. These coefficients of variation incorporate both within-assay and between-assay variability. Daily oestrogen excretion was calculated from the concentration in the urine and the volume of urine collected. Total excretion of oestrone, oestradiol and oestriol was calculated as the sum of these three oestrogens.

### *Statistical analysis*

Oestrogen excretion rates were logarithmically transformed to produce approximately normal distributions, and the mean oestrogen values presented are geometric means. Adjustments of means were made using analysis of covariance for year of urine collection (see below), age (5 year age groups), Quetelet's index (where stated: kg $m^{-2}$) and, in premenopausal women where stated, for stage of menstrual cycle (4 day categories: see below). The geometric means were calculated to describe oestrogen excretion rates in cases and controls. To examine the association of oestrogen excretion with breast cancer risk we used unconditional logistic regression to calculate odds ratios in thirds of the distribution of oestrogen excretion rates in controls, and trend tests for the logarithmically transformed continuous variables. Two-sided $P$-values are quoted.

### *Association of oestrogen excretion with year of blood collection*

To assess whether there was evidence for deterioration of the samples with long-term storage, we examined the association of oestrogen excretion with the year of urine collection (1977–84), adjusting for age and, in premenopausal women, for the stage of the menstrual cycle (see below). In both premenopausal and post-menopausal women there was a statistically significant trend of higher oestrogen excretion in the more recently collected samples. The trends were approximately linear and were similar for the three different oestrogens. The estimated increases were 6.6% total oestrogens per year in premenopausal women and 7.8% total oestrogens per year in post-menopausal women.

To minimise the impact of this effect on the results, year of urine collection was included as a covariate in all subsequent analyses. Adjustment for this variable had very little effect on the case–control comparisons because the average year of urine collection was almost identical in cases and controls.

### Results

### *Characteristics of cases and controls*

Among premenopausal women, cases and controls were similar with respect to age, Quetelet's index, age at menarche and parity; a greater proportion of cases than controls had

**Table I** Characteristics of cases and controls

| Variable | Cases Mean or % | Cases s.d. | Controls Mean or % | Controls s.d. | P[a] |
|---|---|---|---|---|---|
| *Premenopausal* | | | | | |
| *n* | 38 | | 597 | | |
| Age (years) | 41.7 | 4.2 | 41.8 | 4.4 | 0.933 |
| Quetelet's index (kg $m^{-2}$) | 24.4 | 3.4 | 24.7 | 4.0 | 0.676 |
| Age at menarche (years) | 13.1 | 1.5 | 13.1[b] | 1.5 | 0.911 |
| Parous (%) | 87 | | 91 | | 0.629 |
| Oral contraceptives (%)[c] | 74 | | 58 | | 0.078 |
| *Post-menopausal* | | | | | |
| *n* | 31 | | 334 | | |
| Age (years) | 58.3 | 5.7 | 57.8 | 6.2 | 0.669 |
| Quetelet's index (kg $m^{-2}$) | 26.3 | 3.3 | 25.4 | 3.7 | 0.194 |
| Age at menarche (years) | 13.5 | 2.0 | 13.3[d] | 1.4 | 0.503 |
| Parous (%) | 68 | | 79 | | 0.203 |
| Age at menopause (years) | 50.7[e] | 3.4 | 49.4[f] | 3.7 | 0.068 |
| Hormone use (%)[g] | 16 | | 20 | | 0.742 |

[a]Two-sided test for difference between means or proportions. [b]$n=595$. [c]Previous use of oral contraceptives. [d]$n=331$. [e]$n=30$. [f]$n=326$. [g]Previous use of hormones, including hormone replacement therapy but excluding oral contraceptives.

previousy used oral contraceptives but this difference was not statistically significant (Table I). Among post-menopausal women, cases and controls were similar with respect to age, age at menarche and hormone use; cases were slightly fatter, less likely to be parous, and had a later menopause than controls, but these differences were not statistically significant (Table I).

*Urinary oestrogen excretion in premenopausal women*

Table II shows geometric mean total oestrogen excretion in cases and controls, subdivided by the stage of the menstrual cycle at which urine was collected. In both cases and controls total oestrogen excretion was lowest in the early follicular phase (20+ days before the end of the cycle), rising rapidly to a peak at mid-cycle and then falling gradually during the luteal phase. However, the increase in oestrogen excretion at mid-cycle was less in the cases than in the controls. Similar patterns were seen for the individual oestrogens.

Table III shows geometric mean oestrogen excretion in cases and controls, adjusted for the six cycle phase categories used in Table II. Excretion of all three oestrogens was lower in cases than in controls, and total oestrogen excretion was 15% lower ($P=0.136$). Further adjustment for age at menarche, parity and previous use of oral contraceptives did not substantially alter the results (data not shown). Restriction of this analysis to the 34 cases diagnosed more than 2 years after urine collection did not alter this result (geometric mean total oestrogen excretion 15% lower in cases, $P=0.162$; data not shown).

Table IV shows odds ratios for the risk of breast cancer associated with three levels of oestrogen excretion as determined by the tertiles of the distribution in controls. For all three oestrogens risk decreased with increasing oestrogen excretion, but the trends of decreasing risk were not statistically significant.

*Urinary oestrogen excretion in post-menopausal women*

Table III shows geometric mean oestrogen excretion in post-menopausal women. All values are adjusted for year of urine collection and age, but the values are given before and after further adjustments for Quetelet's index. Without adjustments for this variable, geometric mean excretion of the three oestrogens was 20–31% higher in cases than in controls, with total oestrogen excretion 30% higher ($P=0.018$). Adjustment for Quetelet's index slightly reduced these differences. Further adjustment for age at menopause did not substantially alter the results (data not shown). Restriction of this analysis to the 24 cases diagnosed more than 2 years after urine collection did not alter the results (geometric mean total oestrogen excretion, unadjusted for Quetelet's index, was 33% higher in cases, $P=0.022$; data not shown).

One case and two controls had total oestrogen excretion greater than 50 $\mu g$ 24 $h^{-1}$. Exclusion of these three subjects slightly reduced the difference between cases and controls: geometric mean oestrogen excretion, unadjusted for Quetelet's index, was 22% higher in cases, $P=0.056$.

For all three oestrogens the odds ratio for breast cancer was highest in the top third of the distribution (Table IV). These elevations in risk were not statistically significant, but the trend in risk associated with increasing oestrogen excretion was statistically significant for oestradiol ($P=0.022$) and for total oestrogens ($P=0.016$).

### Discussion

The results for premenopausal women do not support the hypothesis that breast cancer risk is increased by high endogenous oestrogen levels, and indeed suggest that average oestrogen excretion might be lower in cases than in

**Table II** Total oestrogen excretion by stage of menstrual cycle in premenopausal cases and controls

| *Days until menstrual period* | | Cases *Geometric mean* | *(95% CI)* | n | Controls *Geometric mean* | *(95% CI)* | n |
|---|---|---|---|---|---|---|---|
| 20+ | Follicular | 12.9 | (5.4–31.0) | 2 | 13.1 | (11.6–14.9) | 95 |
| 16–19 | | 20.2 | (13.7–29.8) | 10 | 23.4 | (20.7–26.4) | 102 |
| 12–15 | Mid-cycle | 25.8 | (14.8–44.7) | 5 | 36.0 | (31.9–40.6) | 104 |
| 8–11 | | 23.1 | (16.4–32.5) | 13 | 29.6 | (26.7–32.8) | 140 |
| 4–7 | Luteal | 18.2 | (7.6–43.5) | 2 | 26.8 | (23.3–30.8) | 78 |
| 0–3 | | 29.6 | (17.9–49.1) | 6 | 24.4 | (21.2–28.1) | 78 |

Values are geometric means, $\mu g$ 24 $h^{-1}$, adjusted for year of urine collection and age (<40, 40–44, 45+ years).

**Table III** Oestrogen excretion in cases and controls

| *Oestrogen* | Cases *Geometric mean* | *(95% CI)* | Controls *Geometric mean* | *(95% CI)* | P[a] |
|---|---|---|---|---|---|
| *Premenopausal*[b] | $n=38$ | | n=597 | | |
| Oestrone | 7.59 | (6.15–9.36) | 8.79 | (8.34–9.27) | 0.192 |
| Oestradiol | 3.95 | (3.19–4.87) | 4.53 | (4.29–4.78) | 0.222 |
| Oestriol | 8.67 | (6.82–11.02) | 10.42 | (9.81–11.07) | 0.149 |
| Total oestrogens | 21.28 | (17.43–25.99) | 24.95 | (23.72–26.24) | 0.136 |
| *Post-menopausal* | $n=31$ | | n=334 | | |
| Oestrone[c] | 1.78 | (1.43–2.22) | 1.48 | (1.38–1.58) | 0.121 |
| Adjusted oestrone[d] | 1.73 | (1.39–2.15) | 1.48 | (1.38–1.58) | 0.185 |
| Oestradiol[c] | 0.98 | (0.79–1.23) | 0.75 | (0.70–0.80) | 0.026 |
| Adjusted oestradiol[d] | 0.96 | (0.77–1.20) | 0.75 | (0.70–0.81) | 0.039 |
| Oestriol[c] | 2.01 | (1.57–2.59) | 1.61 | (1.49–1.74) | 0.101 |
| Adjusted oestriol[d] | 1.92 | (1.51–2.46) | 1.62 | (1.50–1.74) | 0.187 |
| Total oestrogen[c] | 5.21 | (4.26–6.38) | 4.02 | (3.78–4.27) | 0.018 |
| Adjusted total oestrogen[d] | 5.05 | (4.14–6.15) | 4.04 | (3.80–4.29) | 0.036 |

[a]Two-sided test for difference between means. [b]Values are geometric means, $\mu g$ 24 $h^{-1}$, adjusted for year of urine collection, age (<40, 40–44, 45+ years) and day of the menstrual cycle at urine collection (0–3, 4–7, 8–11, 12–15, 16–19, 20+ days before the end of the cycle). [c]Values are geometric means, $\mu g$ 24 $h^{-1}$, adjusted for year of urine collection and age (<55, 55–59, 60+ years). [d]Values are geometric means, $\mu g$ 24 $h^{-1}$, adjusted for year of urine collection, age (<55, 55–59, 60+ years) and Quetelet's index (kg $m^{-2}$).

Table IV Odds ratios (95% confidence intervals) for breast cancer in relation to urinary oestrogen excretion

| Oestrogen | Cut points[a] | Low[b] | Level of oestrogen excretion Middle | High | Trend[c] |
|---|---|---|---|---|---|
| *Premenopausal*[d] | | | | | |
| Oestrone | 6.47, 12.35 | 1.0 | 0.5(0.2-1.2) | 0.4(0.2-1.1) | 0.216 |
| Oestradiol | 3.42, 6.23 | 1.0 | 0.8(0.4-1.8) | 0.4(0.2-1.1) | 0.263 |
| Oestriol | 7.27, 15.19 | 1.0 | 0.7(0.3-1.6) | 0.7(0.3-1.6) | 0.187 |
| Total oestrogens | 18.93, 34.63 | 1.0 | 0.9(0.4-2.0) | 0.5(0.2-1.3) | 0.165 |
| *Post-menopausal*[e] | | | | | |
| Oestrone | 1.15, 1.82 | 1.0 | 0.9(0.3-2.2) | 1.1(0.5-2.8) | 0.111 |
| Oestradiol | 0.61, 0.96 | 1.0 | 0.8(0.3-2.3) | 1.9(0.8-4.6) | 0.022 |
| Oestriol | 1.26, 2.05 | 1.0 | 1.5(0.6-3.9) | 1.8(0.7-4.6) | 0.089 |
| Total oestrogens | 3.21, 4.90 | 1.0 | 0.9(0.4-2.6) | 1.9(0.7-4.7) | 0.016 |

[a]Cut points for levels, $\mu g\ 24\ h^{-1}$. [b]Reference. [c]*P*-value for linear trend for logarithmically transformed continuous variable. [d]Adjusted for year of urine collection, age (<40, 40-44, 45+ years) and day of cycle at urine collection (0-3, 4-7, 8-11, 12-15, 16-19, 20+ days before the end of the cycle). [e]Adjusted for year of urine collection and age (<55, 55-59, 60+ years).

Table V Ratios of oestrogen levels in cases relative to oestrogen levels in controls, divided by phase of cycle

| First author | Date | Sample[a] | Follicular phase Cases | Days | Ratio[b] | Luteal phase Cases | Days | Ratio[b] | Both phases Cases | Ratio[b] |
|---|---|---|---|---|---|---|---|---|---|---|
| Persson | 1964 | U | 15 | – | 1.3 | 8 | – | 1.1 | | |
| Marmorston | 1965 | U | | | | 5 | 18-23 | 0.7 | | |
| Argüelles | 1973 | U | | | | 47 | 14 | 0.7 | | |
| England | 1974 | B | 10 | – | >1 | 10 | – | >1 | | |
| Malarkey | 1977 | B | | | | 5 | – | 0.9 | | |
| Cole | 1978 | U | 73 | 10 | 1.1 | 73 | 21 | 1.1 | | |
| Sherman | 1979 | B | | | | | | | 13 | 1.0 |
| Drafta | 1980 | B | 25 | 12-16 | 1.0 | 25 | 19-24 | 0.3 | | |
| Moore | 1982 | B | | | | | | | 32 | 1.1 |
| MacMahon | 1983 | U | 94 | 10 | >1 | 94 | 21 | >1 | | |
| Bruning | 1985 | B | | | | 17 | 18-24 | 1.1 | | |
| Meyer | 1986 | U | 41 | 6 | 1.0 | 40 | 20-22 | <1 | | |
| Meyer | 1986 | B | | | | 36 | 20-22 | >1 | | |
| Siiteri[c] | 1986 | B | | | | | | | 36 | 1.1 |
| Wysowski[c] | 1987 | B | | | | | | | 17 | 0.8 |
| Bernstein[d] | 1990*a* | B | | | | 39 | 22 | 1.2 | | |
| Bernstein[e] | 1990*b* | B | | | | 42 | 22 | 1.1 | | |
| Zaridze | 1992 | B | | | | | | | 27 | 1.6 |
| Helzlsouer[c] | 1994 | B | 12 | – | 1.2 | 10 | | 0.7 | | |
| Rosenberg[c] | 1994 | B | | | | | – | | 79 | 1.0 |
| Key[f] | 1996 | U | | | | | | | 38 | 0.9 |

[a]U, urine, oestrogen level taken as total oestrogens reported; B, blood (serum or plasma), oestrogen level taken as oestradiol. [b]Ratio of mean value in cases to mean value in controls. [c]Prospective study: the other studies are not prospective. [d]Shanghai Chinese. [e]Los Angeles whites. [f]Current study.

controls. There are too few cases to tell whether there is a different pattern of oestrogen excretion during the menstrual cycle in cases, but our results suggest that baseline levels in cases (early follicular, late luteal) may be similar to those in controls but that cases do not show as large a mid-cycle surge as controls (Table II).

Table V shows the results of other studies (four prospective, 16 case-control) of urinary or plasma oestrogens in premenopausal breast cancer cases and controls, divided according to the stage of the cycle at which samples were collected. The results are expressed as the ratio of the mean value in cases to the mean value in controls. The pattern is not consistent, but it may be noted that for all the samples collected in the follicular phase the ratio of the mean value in cases to that in controls was equal to or greater than one, whereas for the luteal phase samples 6 out of the 14 ratios reported were less than one. The relationship of oestrogen levels in premenopausal women with breast cancer risk is still unclear, but future studies may benefit from considering results in relation to the stage of the menstrual cycle at which samples are collected.

For post-menopausal women, the current study supports the evidence from prior studies suggesting that high oestrogen levels are directly associated with breast cancer risk. The differences between cases and controls were slightly reduced by adjusting for Quetelet's index, as also reported by Toniolo *et al.* (1995). However, because our hypothesis is that high oestrogen levels in post-menopausal women increase breast cancer risk, and that obesity is associated with risk because it is one determinant of oestrogen levels (Grodin *et al.*, 1973; Judd *et al.*, 1982), we do not think that it is appropriate to adjust our results for Quetelet's index. We therefore conclude that high levels of endogenous oestrogens are associated with increased breast cancer risk in post-menopausal women.

One area of potential concern in the current study is the method of selecting the study group of 1000 women for whom assays were completed. As described above, this comprised the sum of two studies planned in 1986, a matched case-control study of breast cancer risk and a study of the effects of cigarette smoking. Treating all these subjects as one study group and conducting an unmatched analysis of oestrogens and breast cancer risk gives the major benefit of increasing the number of cases from 33 to 69. It could be argued that the assembling of controls in two ways might have introduced some bias. There is certainly some overrepresentation of cigarette smoking among the controls (29% current smokers among women with known smoking status in this analysis, compared with 21% current smokers among all women in the Guernsey cohort with known smoking status), but analysis of the relationship of cigarette smoking with urinary oestrogen excretion did not show any differences in total oestrogen excretion (although there was a 19% reduction in excretion of oestriol ($P=0.046$) in post-menopausal smokers; Key *et al.*, in preparation). To investigate whether the structure of the study group may have altered the results we also analysed the data according to the original matching; the results were compatible with those reported here, for example the odds ratios in the top third of the distribution were 0.1, 0.1 and 0.5 for oestrone, oestradiol and oestriol respectively in premenopausal women and 0.7, 1.4 and 1.8 in post-menopausal women, all with wide confidence intervals. We think that it is likely that any disadvantages of the method of assembling the study group are outweighed by the advantage of the much larger number of cases available in the unmatched analysis.

**Acknowledgements**

We thank the following: the women of Guernsey who volunteered for this study; the general practitioners of Guernsey, Dr Bryan Gunton-Bunn, Dr David Jeffs, Miss Louise Davies and the staff at The Greffe for assistance in follow-up; Dr Meg Smith and Ms Marianne Stereff for performing the assays; Mr Graham Clark and Ms Michelle Quinlan for technical help; Miss Lindsey Cutler for preparing the manuscript.

### References

ARGÜELLES AE, POGGI UL, SABORIDA C, HOFFMAN C, CHEKHERDEMIAN M AND BLANCHARDO O. (1973). Endocrine profiles and breast cancer. *Lancet*, **1**, 165–168.

BERNSTEIN L, ROSS RK, PIKE MC, BROWN JB AND HENDERSON BE. (1990*a*). Hormone levels in older women: a study of post-menopausal breast cancer patients and healthy population controls. *Br. J. Cancer*, **61**, 298–302.

BERNSTEIN L, YUAN JM, ROSS RK, PIKE MC, HANISCH R, LOBO R, STANCZYK F, GAO Y-T AND HENDERSON BE. (1990*b*). Serum hormone levels in pre-menopausal Chinese women in Shanghai and white women in Los Angeles: results from two breast cancer case-control studies. *Cancer Causes and Control*, **1**, 51–58.

BINGHAM SA, WILLIAMS R, COLE TJ, PRICE CP AND CUMMINGS JH. (1988). Reference values for analytes of 24-h urine collections known to be complete. *Ann. Clin. Biochem.*, **25**, 610–619.

BROWN JB. (1976). Determination of estriol, estrone and estradiol-17$\beta$ in nonpregnancy urine by spectrophotometry or fluorimetry. In *Methods of Hormone Analysis*, Breuer H, Hamel D and Kruskemper HL (eds) p 446. Wiley: New York.

BRUNING PF, BONFRER JMG AND HART AAM. (1985). Non-protein bound oestradiol, sex hormone binding globulin, breast cancer and breast cancer risk. *Br. J. Cancer*, **51**, 479–484.

COLE P, CRAMER D, YEN S, PAFFENBARGER R, MACMAHON B AND BROWN J. (1978). Estrogen profiles of premenopausal women with breast cancer. *Cancer Res.*, **38**, 745–748.

DRAFTA D, SCHINDLER AE, MILCU ST M, KELLER E, STROE E, HORODNICEANU E AND BALANESCU I. (1980). Plasma hormones in pre- and postmenopausal breast cancer. *J. Steroid Biochem.*, **43**, 793–802.

ENGLAND PC, SKINNER LG, COTTRELL KM AND SELLWOOD RA. (1974). Serum oestradiol-17$\beta$ in women with benign and malignant breast disease. *Br. J. Cancer*, **30**, 571–576.

GARLAND CF, FRIEDLANDER NJ, BARRETT-CONNOR E AND KHAW K-T. (1992). Sex hormones and postmenopausal breast cancer: a prospective study in an adult community. *Am. J. Epidemiol.*, **135**, 1220–1230.

GRODIN JM, SIITERI PK AND MACDONALD PC. (1973). Source of estrogen production in postmenopausal women. *J. Clin. Endocrinol. Metab.*, **36**, 207–214.

HELZLSOUER KJ, ALBERG AJ, BUSH TL, LONGCOPE C, GORDON GB AND COMSTOCK GW. (1994). A prospective study of endogenous hormones and breast cancer. *Cancer Detection Prevention*, **18**, 79–85.

JUDD HL, SHAMONKI IM, FRUMAR AM AND LAGASSE LD. (1982). Origin of serum estradiol in postmenopausal women. *Obstet. Gynecol.*, **59**, 680–686.

KEY TJA AND PIKE MC. (1988). The role of oestrogens and progestagens in the epidemiology and prevention of breast cancer. *Eur. J. Cancer Clin. Oncol.*, **24**, 29–43.

MACMAHON B, COLE P, BROWN JB, PAFFENBARGER R, TRICHOPOULOS D AND YEN S. (1983). Urine estrogens, frequency of ovulation, and breast cancer risk: case-control study in premenopausal women. *J. Natl. Cancer Inst.*, **70**, 247–250.

MALARKEY WB, SCHROEDER LL, STEVENS VC, JAMES AG AND LANESE RR. (1977). Twenty-four-hour preoperative endocrine profiles in women with benign and malignant breast disease. *Cancer Res.*, **37**, 4655–4659.

MARMORSTON J, CROWLEY LG, MYERS SM, STERN E AND HOPKINS CE. (1965). Urinary excretion of estrone, estradiol, and estriol by patients with breast cancer and benign breast disease. *Am. J. Obstet. Gynecol.*, **4**, 460–467.

MEYER F, BROWN JB, MORRISON AS AND MACMAHON B. (1986). Endogenous sex hormones, prolactin, and breast cancer in premenopausal women. *J. Natl. Cancer Inst.*, **77**, 613–616.

MOORE JW, CLARK GMG, BULBROOK RD, HAYWARD JL, MURAI JT, HAMMOND GL AND SIITERI PK. (1982). Serum concentrations of total and non-protein-bound oestradiol in patients with breast cancer and in normal controls. *Int. J. Cancer*, **29**, 17–21.

MOORE JW, CLARK GMG, HOARE SA, MILLIS RR, HAYWARD JL, QUINLAN MK, WANG DY AND BULBROOK RD. (1986). Binding of oestradiol to blood proteins and aetiology of breast cancer. *Int. J. Cancer*, **38**, 625–630.

PERSSON BH AND RISHOLM L. (1964). Oophorectomy and cortisone treatment as a method of eliminating oestrogen production in patients with breast cancer. *Acta Endocrinol.*, **47**, 15–26.

ROSENBERG CR, PASTERNACK BS, SHORE RE, KOENIG KL AND TONIOLO PG. (1994). Premenopausal estradiol levels and the risk of breast cancer. A new method of controlling for day of the menstrual cycle. *Am. J. Epidemiol.*, **140**, 518–525.

SHERMAN BM, WALLACE RB AND JOCHIMSEN PR. (1979). Hormonal regulation of the menstrual cycle in women with breast cancer: effect of adjuvant chemotherapy. *Clin. Endocrinol.*, **10**, 287–296.

SIITERI PK, SIMBERG N AND MURAI J. (1986). Estrogens and breast cancer. *Ann. N.Y. Acad. Sci.*, **464**, 100–105.

TONIOLO PG, LEVITZ M, ZELENIUCH-JACQUOTTE A, BANERJEE S, KOENIG KL, SHORE RE, STRAX P AND PASTERNACK BS. (1995). A prospective study of endogenous estrogens and breast cancer in postmenopausal women. *J. Natl. Cancer Inst.*, **87**, 190–197.

WANG DY, DE STAVOLA BL, BULBROOK RD, ALLEN DS, KWA HG, FENTIMAN IS, HAYWARD JL AND MILLIS RR. (1992). Relationship of blood prolactin levels and the risk of subsequent breast cancer. *Int. J. Epidemiol.*, **21**, 214–221.

WYSOWSKI DK, COMSTOCK GW, HELSING KJ AND LAU HL. (1987). Sex hormone levels in serum in relation to the development of breast cancer. *Am. J. Epidemiol.*, **125**, 791–799.

ZARIDZE D, KUSHLINSKII N, MOORE JW, LIFANOVA Y, BASSALYK L AND WANG DY. (1992). Endogenous plasma sex hormones in pre- and postmenopausal women with breast cancer: results from a case-control study in Moscow. *European Journal of Cancer Prevention*, **1**, 225–230.